# The relationship between having a job and the outcome of brief therapy in patients with common mental disorders

**DOI:** 10.1186/s12888-023-05418-z

**Published:** 2023-12-05

**Authors:** Ard J. van Oosten, Kasper van Mens, Roland W. B. Blonk, Alex Burdorf, Bea Tiemens

**Affiliations:** 1 Parnassia Groep, The Hague, The Netherlands; 2 U-center Epen, Epen, The Netherlands; 3 Altrecht Mental Health Care, Utrecht, The Netherlands; 4 Department of Human Resource Studies, Tilburg University, Tilburg, The Netherlands; 5 Optentia Research Focus Area, North-West University Vaal Triangle Campus, Vanderbijlpark, South Africa; 6 Department of Public Health, Erasmus MC, Rotterdam, The Netherlands; 7 Indigo Service Organization, Utrecht, The Netherlands; 8 Behavioral Science Institute, Radboud University, Nijmegen, The Netherlands; 9 Pro Persona Research, Wolfheze, The Netherlands

**Keywords:** Employment status, Recovery, Brief therapy, Common mental disorders

## Abstract

**Background:**

Previous studies have shown that being employed is associated not only with patients’ health but also with the outcome of their treatment for severe mental illness. This study examined what influence employment had on improvements in mental health and functioning among patients with common mental disorders who received brief treatment and how patients’ diagnosis, environmental and individual factors moderated the association between being employed and treatment outcome.

**Methods:**

The study used naturalistic data from a cohort of patients in a large mental health franchise in the Netherlands. The data were obtained from electronic registration systems, intake questionnaires and Routine Outcome Monitoring (ROM). The International Classification of Functioning, Disability and Health (ICF) framework was used to identify potential subgroups of patients. Logistic regression models were used to analyze the relationship between employment status and treatment outcome and to determine how the relationship differed among ICF subgroups of patients.

**Results:**

A strong relationship was found between employment status and the outcome of brief therapy for patients with common mental disorders. After potential confounding variables had been controlled, patients who were employed were 54% more likely to recover compared to unemployed patients. Two significant interactions were identified. Among patients who were 60 years of age or younger, being employed was positively related to recovery, but this relationship disappeared in patients older than 60 years. Second, among patients in all living situations there was a positive effect of being employed on recovery, but this effect did not occur among children (18+) who were living with a single parent.

**Conclusions:**

Being employed was positively associated with treatment outcome among both people with a severe mental illness and those with a common mental disorder (CMD). The main strength of this study was its use of a large dataset from a nationwide franchised company. Attention to work is important not only for people with a severe mental illness, but also for people with a CMD. This means that in addition to re-integration methods that focus on people with a severe mental illness, more interventions are needed for people with a CMD.

## Background

Being unemployed has a negative impact on both physical and mental health problems [1–5]. A meta-analysis of 237 cross-sectional and 87 longitudinal studies concluded that unemployed individuals have more distress in their lives than employed ones [6]. Employed and unemployed people were found to differ on various indicators of mental health, including mixed symptoms of distress, depression, anxiety, psychosomatic symptoms, subjective well-being, and self-esteem. Also, men and people with blue-collar-jobs were more distressed by being unemployed than women and people with white-collar jobs. Acquiring paid employment alone improves one’s mental and physical health. Participants who were re-employed reported improvement in their general and mental health, physical and social functioning, vitality, bodily pain and role limitations due to emotional or physical problems [7]. A Longitudinal study that included 2,436 employment-aged U.S. adults linked being employed with both the cause and the results of better health. Among both men and women, being employed full-time predicted significantly slower declines in perceived health and physical functioning compared to unemployed individuals. For both sexes, high physical functioning increased the odds of getting or keeping a full-time job [8]. A cross-sectional study using data from 3,857 respondents aged 25 to 55 showed that besides the impact of gender on the prevalence of anxiety and depressive disorders, there was also a gender difference in the impact of work on health. For men, being employed is a significant protective factor against depression and anxiety regardless of whether the man has children. Among women, however, the protective effect of work seems significant only if the woman does not have children. In general, women are more likely to have a depressive or an anxiety disorder than men, but this difference in risk between men and women is decreased among women who have a job [9].

The relationship between unemployment and physical and mental health problems has been confirmed not just through self-reports of participants, but has also been confirmed in other ways such as a prospective cohort study on the relationship between unemployment and the risks for acute myocardial infarction [10] and a review of the worldwide literature on unemployment and the risk of suicide [11].

Several studies have shown that being employed is associated not only with perceived health and mental and physical functioning, but also with the treatment outcome of patients with mental health problems [12–15]. In a naturalistic sample of 917 outpatients in psychiatric specialty care who had a panic disorder with or without (a) agoraphobia, (b) agoraphobia without panic, (c) social phobia, or (d) generalized anxiety disorder, not having a regular job predicted a decreased likelihood of a positive treatment response [13]. In a sample of 180 depressed outpatients who were randomized to receive cognitive therapy and antidepressant medication, those who were moderately to severely depressed had a better response to cognitive therapy relative to antidepressant medication if they were unemployed [14]. Lorenzo-Luaces et al. developed prognostic indices to help guide the selection of treatments that differed in intensity among treatment as usual, treatment that started with a low-intensity (brief therapy), and treatment that started with a high-intensity (cognitive-behavioral therapy). They found that being unemployed predicted a lower likelihood of recovery for depressed patients [15].

Within mental health care, research on the impact of work on recovery has focused mainly on people with severe mental illness [32–34]. In this group of patients, there is also an increasing focus on employment status [35]. This attention is much less in patients with common mental disorders. Thus, in line with the studies of Schat et al., Fournier and DeRubeis and Lorenzo-Luaces et al. [13–15], which focused on specific diagnostic groups, the question remains as to whether the positive association between having work and treatment outcome in patients with severe mental disorders also applies to patients with a variety of different common mental disorders. If it does, does the relationship occur among all patients, or can subgroups be identified in which the association is more or less strong than among other subgroups? For example, are there differences based on health-related factors such as diagnosis; employment factors, such as whether the work is full-time or part-time; and individual factors, such as patients’ age or gender.

The International Classification of Functioning (ICF) framework can be used to identify the main factors that can distinguish among the possible subgroups [16]. The ICF is a classification of health and health-related domains, and it focuses on the functioning of persons in their context. The ICF conceptualizes how patients’ health status, functions/structures, activities, participation, and environmental and personal factors are interrelated and influence one another. It is a biopsychosocial model of disability. The assumption is that biological, psychological and social factors all play a role in the development of impairments, activity limitations and participation restrictions. The ICF was officially endorsed by all 191 WHO Member States in the Fifty-fourth World Health Assembly on 22 May 2001 as the international standard to describe and measure health and disability [17].

**Fig. 1:**
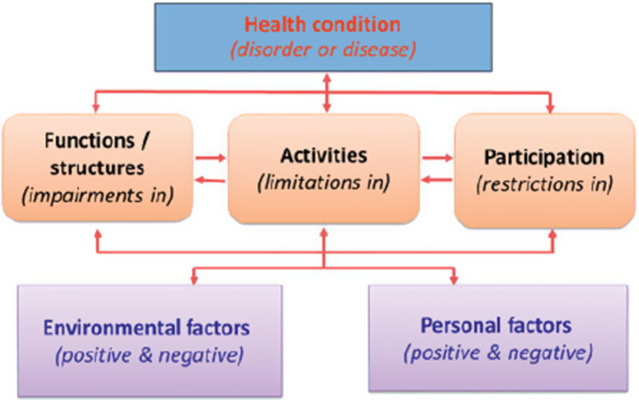
ICF scheme

In the ICF diagram (Fig. 1), being employed is considered to be an environmental factor. Treatment outcome is viewed as resulting from changes from baseline in patients’ health condition, functions/structures, activities, participation, personal factors and their environment.

Several studies have further investigated the interrelationships among the different elements in the ICF model. In a scoping review, evidence was found that common mental disorders, greater symptom severity, co-morbidity (health conditions), heavy job demands, low job control, high job strain (environmental factors), female gender, lower educational level, smoking behaviour and low perceived general health (personal factors) are all predictors of absence due to illness (participation factor) [18]. An earlier return to work (i.e., participation) is associated with lower symptom severity (health condition), younger age, and positive expectations concerning sick-leave duration (personal factors).

The British Household Panel Survey (BHPS) is an on-going annual panel survey of a representative sample of more than 5,000 households across Great Britain [19]. Results from Waves 1 to 8 confirmed the relationships and influences depicted in the ICF framework. A lower chance of recovery was associated with poor physical health (health condition); being female and older (personal factors); marital transition due to the death of a partner or through separation or divorce; remaining unemployed, loss of a job, little social support and caring for a sick relative (environmental factors). Severity of the disorder (health condition) was associated with lower likelihood of recovery and a longer time in recovery. Greater chances of recovery were observed in those who obtained a job or were married (environmental factors) and those who reported being socially engaged.

### Aims of the study

The present study examined (a) the influence of employment on improvements in mental health and functioning among patients with common mental disorders and (b) how patients’ health, environmental and individual factors moderated the association between being employed and mental health and functioning.

## Methods

### Design and setting

The study used naturalistic data from a cohort of patients in a large mental health franchise in the Netherlands. The franchise has about 200 locations across a large part of the Netherlands, including both urban and rural areas. It offers outpatient brief therapy for patients with mild to moderate complex psychological problems. The patient population is treated in basic mental healthcare, which is a division of the Dutch mental healthcare system between general practitioners and specialized mental healthcare. The financing of basic mental healthcare includes four levels of treatment: short, medium, intensive and chronic. After intake, patients are allocated to one of the treatment levels based on their need for care and the type and severity of their symptoms. Basic mental health treatments are relatively short, with the most intensive level including an average of 12 sessions during a period of about six months. The treatments are transdiagnostic, focusing on a specific problem such as ruminations, or they are a short protocolized treatment for a specific disorder, such as PTSD, or they include a specific modality, such Eye Movement Desensitization and Reprocessing (EMDR).

### Procedure

#### Data sources

Data were obtained from an anonymized dataset. Data from this dataset came from electronic patient records and questionnaires administered as a part of Routine Outcome Monitoring (ROM). ROM was used during the treatment to determine whether the patient was responding well to treatment. Data from adult patients (≥ 18 years, *N* = 91,959) whose treatment had started in the period from 2014 to 2018 were included in the dataset. Patients in the chronic treatment category were excluded. From this set of patients, only patient records with valid ROM measures before and after treatment were included, and we excluded patients who were already partially in remission, resulting in a dataset with 14,072 records.

### Measures

#### Outcome questionnaire 45.2

The Outcome Questionnaire (OQ-45.2) [20] was part of the ROM in usual care. It is intended to measure three domains of functioning: symptom distress (SD), interpersonal relationships (IR) and social role (SR) performance. The OQ-45.2 included 45 items that are scored on a five-point Likert scale, ranging from *never* (*0*) to *almost always* (*4*). The SD subscale includes 25 items that are associated with the most common disorders seen in public mental health care: depression, anxiety and addiction to alcohol or drugs. The IR subscale consists of 11 items that measure the patient’s functioning in the relationship with his or her partner, family and friends. The SR subscale contains nine items that measure functioning in school, work and leisure activities. There are nine items that are reverse-scored. The psychometric properties of the Dutch OQ-45 have been found to be adequate [20].

### Treatment outcome

Primary treatment outcome was defined as the degree of symptom improvement, as measured by the symptom distress scale of the Outcome Questionnaire (OQ-45.2) [20]. Treatment outcome was defined as the difference between pre- and post-treatment scores on the OQ-45.2; these scores were dichotomized as *recovered* and *not recovered*. *Recovered* was defined as an improvement of 10 or more points on the SD scale of the OQ-45.2 (based on Jacobson & Truax’s criteria for reliable change [21] and a final score < 33.

### Environmental factors

#### Employment

At the start of the treatment, a questionnaire was administered which asked about certain aspects of the patient’s current employment. Two variables were created. First, in order to determine whether the hypothesized relationship between employment and treatment outcome occurred, employment was defined as a dichotomous variable (employed versus unemployed). Second, the number of days worked per week was indicated as an ordinal variable that ranged from *0* to *7*. The purpose was to determine how number of days worked per week was related to treatment outcome.

#### Living condition, marital status and urbanization level

Factors related to the home environment, which were included in our observational dataset, play a role in the ICF scheme. Specifically, the variables *living condition*, *marital status* and *urbanization level* were created as categorical variables. Urbanization level was defined on a five-point ordinal scale based on the patient’s postal code; the endpoints of the scale indicate whether the patient lived in a more rural area (*1* on the scale) of a more urban area (*5* on the scale).

### Clinical factors

Four clinical factors were evaluated as potential confounders: treatment intensity, treatment duration in hours, treatment duration in days, and geographical location of the clinic(s). The treatment intensity (short, medium, intensive) is linked to the reimbursement structure. Based on patients’ intake and pre-treatment scores from the Outcome Questionnaire, their therapist allocated them to one of the three intensities. Treatment intensity was hypothesized to influence patients’ chances of recovery because the intensity allocated is associated with the type and severity of the patients’ symptoms. Treatment duration in hours is the total number of hours that a healthcare professional spends on a patient, which we hypothesized would influence patients’ chances of recovery. Treatment duration was calculated as the number of days between the start and completion of a treatment. Treatment duration is related to the frequency of patients’ visits and could therefore influence the chances of recovery. Moreover, a very short duration might indicate a treatment drop-out. The location of clinics was included as a potential confounder because even though the organization has a common way of dealing with patients, there might still be local differences.

### Personal factors

Four personal factors were included: *gender, age, socio-economic status* and *country of origin.* When the study was conducted, gender (male or female) was designated as a dichotomous variable, but currently we designate gender more broadly. Age was a categorical variable based on the different decades of life (< 30, 30–40, 41–50, 51–60, and > 60 years). The categories that we used are based on the different phases of the life cycle [22].

Socio-economic status was a continuous variable that ranged from *− 6* (*very low*) to *3* (*very high*). The variable was constructed with the use of a table called *Statistics Netherlands*, which indicates the average socio-economic status of people within each postal code. Country of origin was a categorical variable based on the nation of birth of the patients’ parent(s). It indicates whether the patient was an immigrant (i.e., whether one or both parents were born in the Netherlands or another country).

### Health condition

**Fig. 2:**
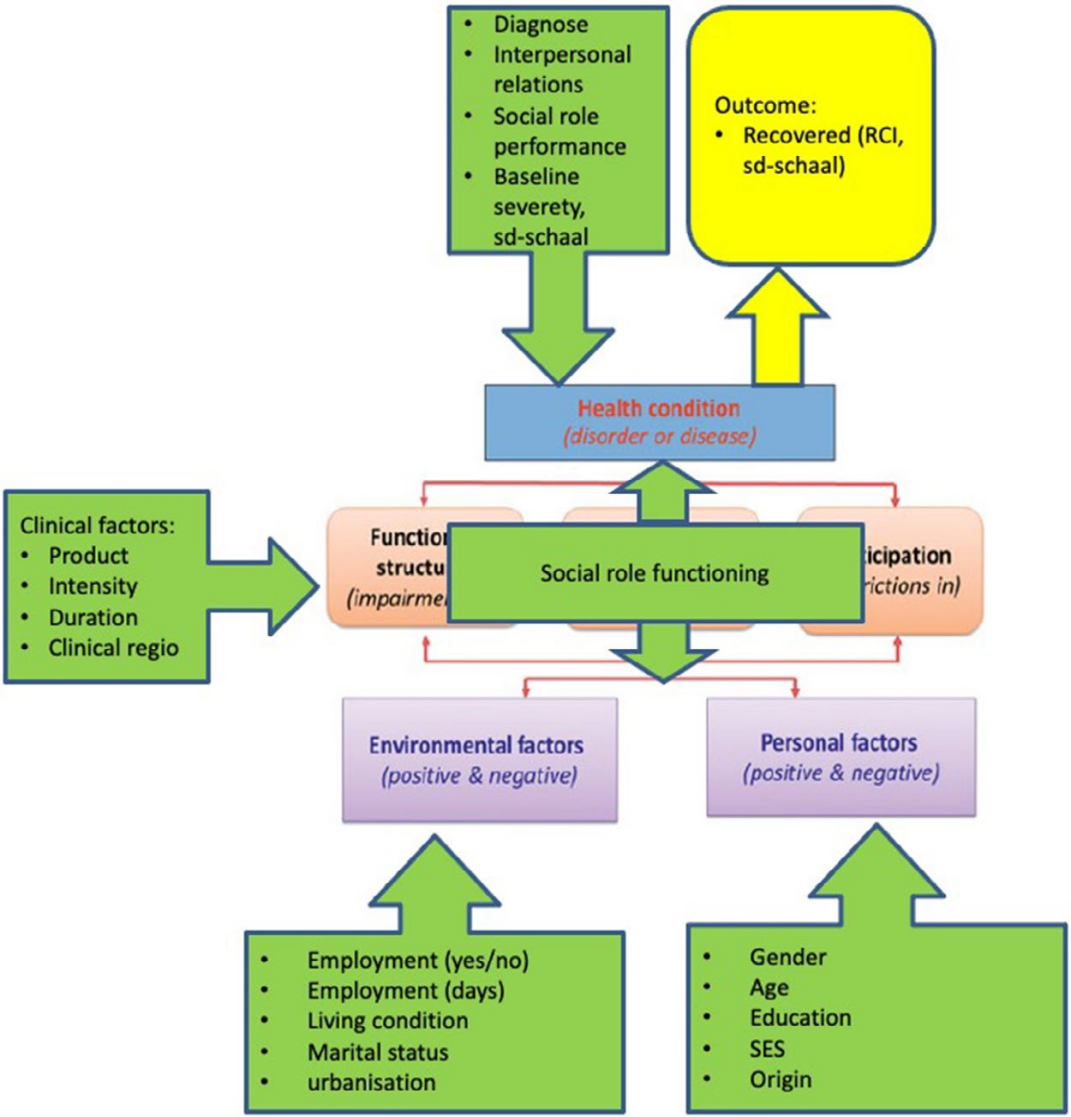
The factors analyzed in the ICF scheme

The ICF scheme takes into account such medically relevant factors as the patient’s diagnosis, interpersonal relationships, social role performance and the baseline severity of symptoms and overall health condition. The variable *diagnostic group* was created as a categorical variable based on patients’ symptoms and the DSM-IV classification of mental disorders [23]. Interpersonal relationships (IR) and social role performance (SR) were created as dichotomous variables based on patients’ pre-treatment scores on the OQ-45.2 and the cut-off scores indicative of low clinical functioning (IR > 15, SR > 12) [24]. These variables were dichotomized in order to improve interpretability of relationships between patients’ health condition and their employment status and recovery. *Baseline severity* was created as a continuous variable and was included as a potential confounding variable in patients’ chances of recovery. Figure 2 depicts the variables that were analyzed in the study.

### Statistical analyses

Logistic regression models were used to examine the relationship between employment and improvements in mental health and functioning in patients with common mental disorders, as well as how patients’ health, environmental and individual factors moderated the association between being employed and mental health and functioning. A cut-off *p*-value of 0.05 was used to indicate statistical significance. All of the statistical analyses were performed using R, an open-source statistical programming environment [25]. A step-by-step approach was used to construct the final model. The analyses included the following steps:

### Step 1

In the first step, the first research aim was addressed, and a model (1.1) with employment as a dichotomous variable was constructed. Subsequently, another model was created that included number of working days (1.2). Models 1.1 and 1.2 were compared using the Akaike Information Criterion (AIC) [26] as a measure to compare how well different models fit the observational data. A lower AIC indicates a model with better fit. The model with the lowest AIC was used as Model 1.1 in subsequent steps.

### Step 2

In the second step of the analyses, potential confounding variables related to patients’ clinical factors and their health condition were assessed to determine whether they altered the relationship between employment status and the chances of recovery. Specifically, the clinical factors and health condition variables were added to Model 1.1 to determine whether they were significant and the extent to which they modified the association between employment status and treatment outcome. The contribution of these factors to the association between employment status and treatment outcome was expressed as the percentage of reduction in the OR employment status and outcome. The significant clinical variables were retained and used in consecutive steps in Model 2.

### Step 3

In the third step of the analyses, the relationship between recovery, employment status, and each of the personal and environmental factors was explored. In Model 3, all of the environmental and personal variables were added to Model 2.

### Step 4

In Step 4, the second research aim was evaluated by identifying the interactions between employment status and the significant variables from Step 3 in Model 4. Only the significant interactions are shown in the Results section.

## Results

Data from 14,072 patients in Dutch mental healthcare were analyzed. 5,682 (40.38%) of these patients, whose treatment had started from 2014 to 2018, recovered. To assess the influence of patients’ work status on their likelihood of recovery, a stepwise approach was used to build regression models. The various factors from the International Classification of Functioning (ICF) were used to analyze their association with employment and mental health functioning. The results are displayed in Table 1, Model 1.1, and Model 1.2.

**Table 1:** Regression output step 1, influence of employment on the odds of recovery

Variable	Category	N (%)	OR (95% CI)	*p*-value
**Model 1.1**				
Employment	No	4679 (33.3)	Reference group	
	Yes	9393 (66.7)	1.72 (1.60–1.85)	< 0.001
**Model 1.2**				
Number of working days	0	4668 (33.2)	Reference group	
	1	304 (2.2)	1.73 (1.37–2.19)	< 0.001
	2	624 (4.4)	1.90 (1.60–2.25)	< 0.001
	3	1461 (10.4)	1.84 (1.64–2.08)	< 0.001
	4	2297 (16.3)	1.73 (1.56–1.92)	< 0.001
	5	4069 (28.9)	1.70 (1.55–1.85)	< 0.001
	6	240 (1.7)	1.58 (1.21–2.06)	< 0.001
	7	325 (2.3)	1.43 (1.13–1.79)	0.002
	Unknown	84 (0.6)	1.46 (0.93–2.25)	0.09

As shown in Table 1, the results of the regression analysis of the dichotomous employment variable were significant (*p* < .05). Having a job was positively related to patients’ recovery. The results of the regression analysis of number of working days were also significant (*p* < .05). Patients who worked two days a week had the greatest chance of recovery, and patients who worked seven days a week had the lowest chance of recovery, although the ratio was still 1.43, compared to having no job at all. All of the confidence intervals for the number of working days overlapped, indicating that there were no significant differences among the number of days worked (except when working days were compared to not having worked at all).

The Akaike Information Criterion (AIC) [26] in Model 1.1 was 18,773 with a residual variance of 18,769 and 14,070 degrees of freedom. In Model 1.2, AIC was 18,779 with a residual variance of 18,761 and 14,063 degrees of freedom. Because the AIC in Model 1.1 was lower than in Model 1.2, it was preferred and was used in the subsequent steps. These results are shown in Table 2 and Model 2. Table 2 shows a small decrease in the coefficient for employment (from 1.72 to 1.62) when the clinical and health-related variables were added to Model 1.1. This was a 7% reduction in the OR. All the variables in the model were significant (*p* < .05), except when *depression* diagnosis (*p* = .23) was compared with *anxiety*. The AIC in this model was 17,735 with a residual variance of 17,711 and 14,060 degrees of freedom.

**Table 2:** Regression output of step 2, influence of clinical factors and health condition

Variable	Category	N (%)	OR (95% CI)	*p*-value
**Model 2**				
Employment	No		Reference group	
	Yes	9393 (66.7)	1.62 (1.50–1.75)	< 0.001
Treatment intensity	Light	661 (4.7)	Reference group	
	Medium	3930 (27.9)	1.51 (1.27–1.80)	< 0.001
	Intensive	9123 (64.8)	1.40 (1.17–1.67)	< 0.001
	Unknown	358 (2.5)	0.51 (0.38 – 0.69)	< 0.001
Intensity in hours		10.55 (4.3)	0.92 (0.91 – 0.94)	< 0.001
Duration in days		164.60 (83.79)	1.00 (1.00–1.00)	< 0.001
Clinical region	1	3864 (27.5)	Reference group	
	2	2566 (18.2)	1.09 (0.97–1.21)	0.14
	3	7642 (54.3)	1.17 (1.08 – 1.28)	< 0.001
Diagnosis	Anxiety	5291 (37.6)	Reference group	
	Depression	5916 (42.0)	0.95 (0.88–1.03)	0.23
	Other	2865 (20.4)	0.72 (0.65 – 0.79)	< 0.001
Baseline severity		52.67 (11.63)	0.96 (0.96 – 0.97)	< 0.001

In Step 3, each of the environmental and personal variables was added to Model 2. The resulting Model 3 is shown in Table 3. The AIC for Model 3 was 17,598 with a residual variance of 17,530 and 14,038 degrees of freedom. Table 3 shows that when employment status was combined with other variables, it was still significant (OR = 1.58, *p* < .001). Moreover, patients’ age was negatively related to their chances of recovery. That is, patients who were older than 40 had significantly lower chances of recovery compared to patients who were younger than 30. High or low functioning in social roles was not significantly related to treatment outcome; however, low functioning in interpersonal relationships had a significant negative effect (OR = 0.66, *p* < .001). Two of the living arrangements had a positive effect compared to living alone; they were *with partner* (OR = 1.15, *p* = .04) and *child living with single parent* (OR = 1.23, *p* = .03). Not being married compared to being married had a significant positive effect (OR = 1.17, *p* = .04). Living in urban areas *3* or *4* had a positive effect; OR = 1.20, *p* < .001, and OR = 1.16, *p* < .03, respectively. Lastly, patients’ sex and their socio-economic status were not significantly associated with their recovery.

**Table 3:** Regression output of step 3, relations between personal and environmental factors and recovery^a^

Variable	Category	N (%) or mean (sd)	OR (95% CI)	*p*-value
**Model 3**				
Employment	No		Reference group	
	Yes	9393 (66.7)	1.58 (1.45–1.71)	< 0.001
Gender	Male		Reference group	
	Female	9128 (64.9)	1.06 (0.98–1.14)	0.17
Age Category	< 30	4457 (31.7)	Reference group	
	30–40	3376 (24.0)	0.92 (0.83–1.03)	0.13
	40–50	2683 (19.1)	0.78 (0.69 – 0.88)	< 0.001
	50–60	2342 (16.6)	0.71 (0.63 – 0.81)	< 0.001
	60+	1214 (8.6)	0.77 (0.66 – 0.90)	< 0.001
Social Role	High functioning		Reference group	
	Low functioning	11,763 (83.6)	1.03 (0.93–1.14)	0.59
Interpersonal Relations	High functioning		Reference group	
	Low functioning	11,026 (78.4)	0.66 (0.60–0.72)	< 0.001
Living condition	Single	2822 (20.1)	Reference group	
	Single parent	1033 (7.3)	0.97 (0.82–1.15)	0.74
	With partner	2609 (18.5)	1.15 (1.01–1.30)	0.04
	With partner and children	3593 (25.5)	1.08 (0.94–1,25)	0.26
	Child (18+) with single parent	305 (2.2)	1.23 (0.94–1.58)	0.03
	Child (18+) with parents	682 (4.8)	1.22 (1.02–1.49)	0.13
	Other	595 (4.2)	1.02 (0.84–1.24)	0.82
	Unknown	2433 (17.3)	0.23 (0.03–1.03)	0.08
Marital Status	Married	6062 (43.1)	Reference group	
	Not married	4098 (29.1)	1.14 (1.01–1.28)	0.04
	Divorced / Widowed	1476 (10.5)	1.05 (0.91–1.22)	0.48
	Unknown	2436 (17.3)	2.84 (0.62–20.41)	0.22
SES		− 0.86 (1.23)	1.00 (0.97–1.03)	0.85
Urbanisation	1	4595 (32.7)	Reference group	
	2	3944 (28.0)	1.06 (0.96–1.17)	0.24
	3	2980 (21.2)	1.20 (1.08–1.33)	< 0.001
	4	1631 (11.6)	1.16 (1.02–1.33)	0.03
	5	922 (6.6)	1.00 (0.86–1.17)	0.96

^a^ Adjusted for the confounding variables in model 2

**Fig. 3:**
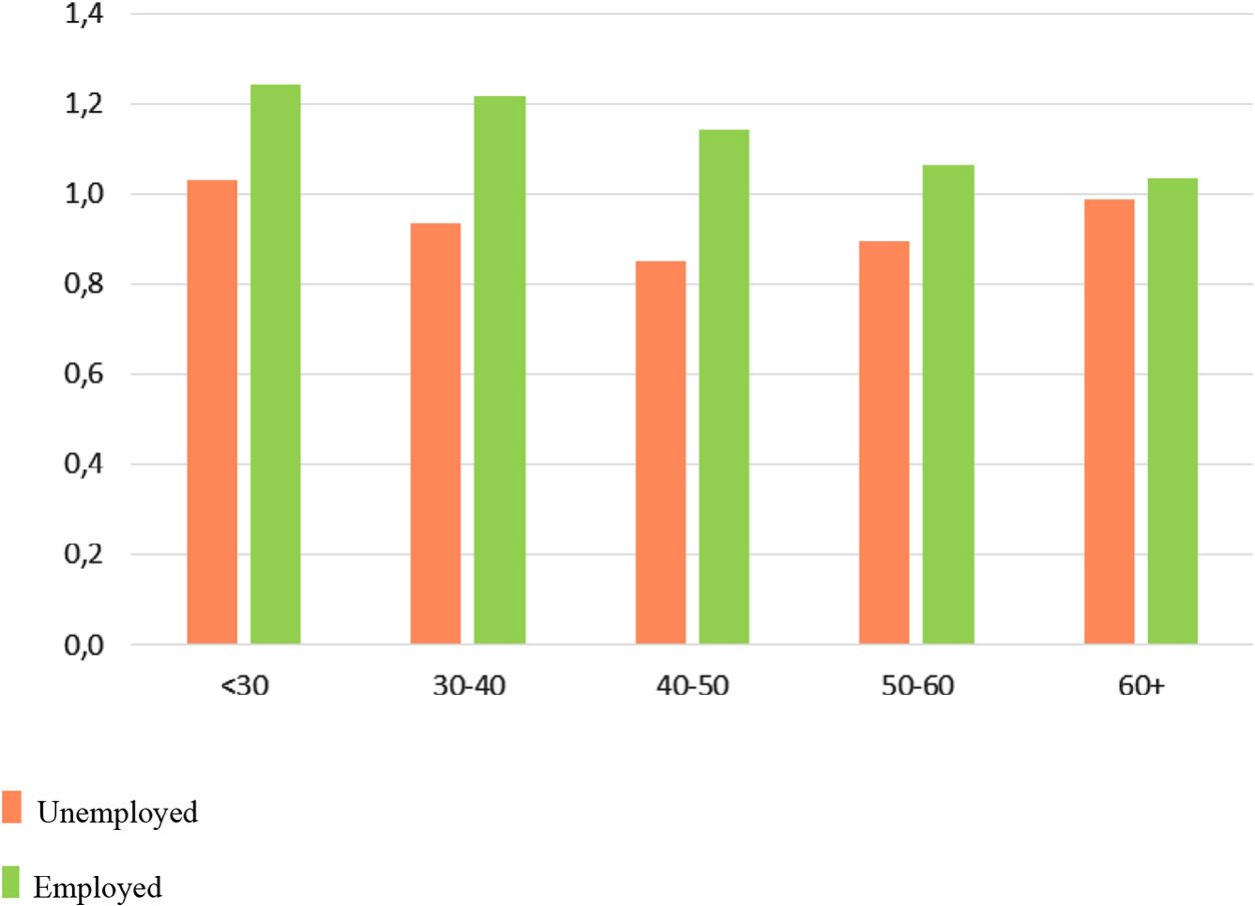
Odds ratios of recovery for employment/unemployment and age category

**Fig. 4:**
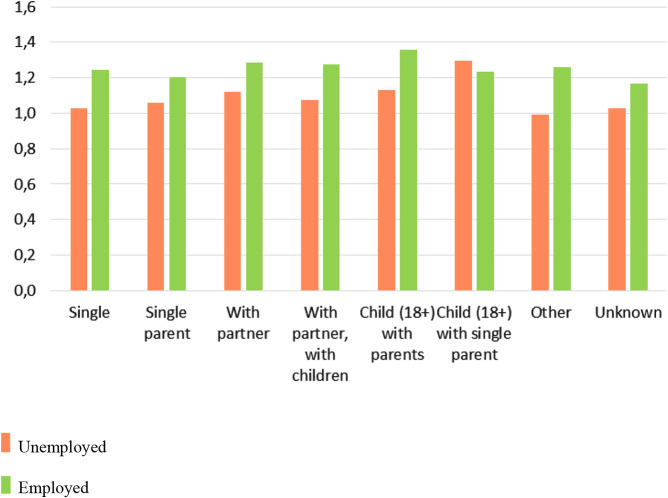
Odds ratios of recovery for employment/unemployment and living situation

The last step in the analysis involved assessing the interactions between employment status and the significant variables from Step 3 on patients’ recovery. The results in Table 4 show that employment status was still significant (OR = 1.54, *p* < .001), although the OR decreased by another 5% (from 1.62 to 1.54). Patients’ age was negatively related to their chances of recovery, so that patients who were between 30 and 60 years old had a significantly lower chance of recovery compared to patients who were younger than 30. High vs. low functioning in social roles was not significant. Low functioning in interpersonal relationships was no longer significantly negatively related to treatment outcome. Only one of the living arrangements had a positive effect compared to adults living alone; namely, it was *child (18+) living with parents* (OR = 1.70, *p* = .01). Both (a) *being married* compared to *not being married* and (b) *living in urban areas 3 or 4* no longer had a significant effect. Finally, as depicted in Figs. 3 and 4, interactions were found (a) between patients’ age and employment status and their chances of recovery and (b) between their living arrangements and employment status and chances of recovery. *First*, among patients who were younger than 60, there was a positive effect of being employed on recovery, but this effect did not occur among patients who were older than 60. *Second*, among patients in all living situations there was a positive effect of being employed on recovery, but this effect did not occur among children (18+) who were living with their single parent.

**Table 4:** Regression output of significant interaction variables of step 4^a^

Variable	Category	N (%) or mean (sd)	OR (95% CI)	*p*-value
**Model 4**				
Employment	No		Reference group	
	Yes	9393 (66.7)	1.54 (1.18–2.02)	< 0.01
Age Category	< 30	1505 (32.2)	Reference group	
	30–40	826 (17.7)	0.80 (0.65 – 0.99)	0.04
	40–50	782 (16.7)	0.64 (0.51 – 0.80)	< 0.01
	50–60	785 (16.8)	0.72 (0.57 – 0.89)	< 0.01
	60+	781 (16.7)	0.91 (0.72–1.16)	0.45
Living condition	Single	1117 (23.9)	Reference group	
	Single parent	426 (9.1)	1.07 (0.82–1.40)	0.60
	With partner	822 (17.6)	1.22 (0.96–1.56)	0.10
	With partner and children	893 (19.1)	1.10 (0.84–1.44)	0.48
	Child (18+) with parents	273 (5.8)	1.24 (0.91–1.69)	0.16
	Child (18+) with single parent	129 (2.8)	1.70 (1.14–2.52)	0.01
	Other	240 (5.1)	0.92 (0.66–1.26)	0.61
	Unknown ^b^	779 (16.6)		
Employment * Age Category	< 30	2952 (31.4)	Reference group	
	30–40	2550 (27.1)	1.19 (0.94–1.51)	0.159
	40–50	1901 (20.2)	1.29 (0.99–1.67)	0.059
	50–60	1557 (16.6)	0.97 (0.75–1.27)	0.836
	60+	433 (4.6)	0.72 (0.52 – 0.9980)	0.049
Employment * Living condition	Single	1705 (18.2)	Reference group	
	Single parent	607 (6.5)	0.87 (0.62–1.21)	0.41
	With partner	1787 (19.0)	0.89 (0.67–1.18)	0.42
	With partner and children	2700 (28.7)	0.97 (0.71–1.34)	0.87
	Child (18+) with parents	409 (4.4)	0.99 (0.67–1.46)	0.97
	Child (18+) with single parent	176 (1.9)	0.58 (0.34–0. 96)	0.04
	Other	355 (3.8)	1.12 (0.75–1.68)	0.57
	Unknown ^b^	1654 (17.6)		

^a^ Adjusted for the confounding variables in Model 2

^b^ The calculation of OR and CI for this category was not possible due to correlation with the unknown category in marital status

## Discussion

The results of this study support previous research, which found that being employed was positively associated with patients’ treatment outcome. In fact, among the patients from the Netherlands with common mental disorders, there was a strong relationship between being employed and recovery. There were only small differences between patients working part-time and those working full-time in the association between employment status and recovery. These differences were not significant. We conclude that being employed is most important, regardless of whether it is full-time or part-time. Only patients’ age and living situation moderated the relationship between being employed and recovery.

Interactions were found between patients’ age and employment status and their recovery. Among patients who were 60 years old or younger, there was a positive relationship between being employed and recovery, but this effect did not occur among patients older than 60. We suggest that having a job for people aged 60 and older no longer promotes recovery because aging changes the personal circumstances of those affected. Descriptive studies show poorer functional outcomes and lower quality of life among older adults because severe mental illnesses are strongly associated with social isolation, depression, cognitive impairment and chronic medical conditions [27]. These changes are apparently not compensated for by work. Marie Jahoda’s latent deprivation model might further explain why having a job for those aged 60 and older no longer promotes recovery [28]. Aging can lead to a lack of the manifest reason for working (to earn money), but also to a lack of the five so-called latent functions of work: Time structure, social contacts, collective purpose (i.e., feeling useful to other people), status and activity. Finally, Deci and Ryan’s self-determination theory might explain why older people benefit less from work than younger people [29]. This theory states that work contributes positively to recovery only if it fulfills the three innate psychological needs: competence, autonomy and connectedness. If a person’s efforts at work are no longer valued, and his or her autonomy and connectedness at work diminish, this can lead to a decrease in mental health.

Among patients in all living situations there was a positive effect of being employed on recovery, but this effect did not occur among children (18+) who were living with a single parent. The lack of an effect among these patients might occur because the tasks that are required of them in caring for their single parent hinder their recovery. Meta-analyses of the physical and mental health effects of informal care have shown higher levels of depression and physical health problems in informal caregivers compared to non-caregivers [30, 31]. Work apparently has insufficient protective effects for these patients and might instead be an additional burden.

With regard to gender differences in recovery, Plaisier et al. [9] found that the relationship between employment status and mental illness differed among males and females. We, therefore, expected to find a similar relationship between gender and recovery, but this effect did not occur.

Our research findings have implications for the focus of reintegration interventions. Until now, reintegration has focused mainly on people with severe mental illness and a lot of research has been conducted on Individual Placement and Support (IPS), a vocational rehabilitation programme that was developed in the United States to improve employment outcomes for people with severe mental illness [32–34]. Readjustment is necessary because employment is also important in the recovery of patients with common mental disorders. By educating their patients about the positive effects of work on mental health and recovery, including the topic of work in their treatment plan and supporting patients to stay at work or return to work, clinicians can further contribute to the mental health of people with common mental disorders [35].

### Strength and limitations

The main strength of this study was its use of a large dataset from a nationwide franchised company. A large data set enables more accurate and robust analysis. By using data from a large sample of clients, it was possible to assess the interactions of potential moderators with the association between work and treatment outcome. Because this large dataset was available, we are able to generalize findings to a larger population.

There are, however, some limitations that should be acknowledged. For instance, because we used naturalistic data, we cannot exclude the possibility that natural selection mechanisms were involved. A further limitation is that only 15% of the patients in the sample could be included in the analyses because for the remainder of the sample, the values for some of the variables were not available.

## Conclusions

The present results indicate that the association between employment status and the outcome of specialty treatment for patients with mental disorders can be generalized to the treatment of patients with common mental disorders with the use of brief therapy. Being employed was positively associated with patients’ recovery. In summary, a strong relationship was found between paid employment and improvements in mental health and functioning among patients with common mental disorders.

Two significant interactions among the variables related to patients’ recovery were identified. Among patients who were 60 years of age or younger, being employed was positively related to recovery, but this effect did not occur in patients older than 60 years. Second, among patients in all living situations there was a positive effect on recovery of being employed, but this effect did not occur among children (18+) who were living with a single parent.

In the day-to-day practice of treating people with common mental disorders, it is often unknown to the therapists whether patients are employed, and if they are, whether they have been absent from work, and whether there are problems at work that impede the patients’ recovery, e.g., bullying or other work-related conflicts. Clinicians and patients are often unaware of the positive impact of work on patients’ recovery. The findings from this study underscore the importance of starting a conversation with patients about work and to take patients’ work into consideration during the treatment.

## Data Availability

The datasets used and/or analysed during this study are available from the corresponding author on reasonable request.

## Declarations

### Methods

All methods were carried out in accordance with relevant guidelines and regulations.

## References

[1] Waddell G, Burton AK. Is work good for your health and well-being? London: The Stationery Office; 2006. https://www.gov.uk/government/publications/is-work-good-for-your-health-and-well-being. Accessed April 1 2023.

[2] Gill F (1999). The meaning of work: lessons from sociology, psychology, and political theory. J Socio Econ.

[3] Van Hedel K, Van Lenthe FJ, Avendano M, Bopp M, Esnaola S, Kovacs K (2015). Marital status, labour force activity and mortality: a study in the USA and six European countries. Scand J Public Health.

[4] Affleck W, Carmichael V, Whitley R (2018). Men’s mental health: social determinants and implications for services. Can J Psychiatry.

[5] Dalglish SL, Melchior M, Younes N, Surkan PJ (2015). Work characteristics and suicidal ideation in young adults in France. Soc Psychiatry Psychiatr Epidemiol.

[6] Paul KI, Moser K (2009). Unemployment impairs mental health: meta-analyses. J Vocat Behav.

[7] Schuring M, Mackenbach J, Voorham T (2011). The effect of re-employment on perceived health. J Epidemiol Community Health.

[8] Ross CE, Mirsky J (1995). Does Employment affect Heath?. J Health Soc Behav.

[9] Plaisier I, de Bruijn JGM, Smit JH (2008). Work and family roles and the association with depressive and anxiety disorders: differences between men and women. J Affect Disord.

[10] Dupre ME, George LK, Liu G, Peterson ED (2012). The cumulative effect of unemployment on risks for Acute Myocardial Infarction. Arch Intern Med.

[11] Haw C, Hawton K, Gunnell D, Platt S (2015). Economic recession and suicidal behaviour: possible mechanisms and ameliorating factors. Int J Soc Psychiatry.

[12] Tiemens B, Böcker K. KloosM. Prediction of treatment outcome in daily generalized mental healthcare practice: first steps towards personalized treatment by clinical decision support. Eur J Person Centered Healthc, 2016: 24–32.

[13] Schat A, Van Noorden MS, Noom MJ (2013). Predictors of outcome in outpatients with anxiety disorders: the Leiden routine outcome monitoring study. J Psychiatr Res.

[14] Fournier J, DeRubeis R (2009). Prediction of response to medication and cognitive therapy in the treatment of moderate to severe depression. J Consulting Clin Psychol.

[15] Lorenzo-Luaces L, DeRubeis RJ, van Straten A (2017). A prognostic index (PI) as a moderator of outcomes in the treatment of depression: a proof of concept combining multiple variables to inform risk-stratified stepped care models. J Affect Disord.

[16] Heerkens YF, de Weerd M, Huber M, de Brouwer CPM (2017). Reconsideration of the scheme of the international classification of functioning, disability and health: incentives from the Netherlands for a global debate. Disabil Rehabil.

[17] https://www.who.int/standards/classifications/international-classification-of-functioning-disability-and-health. Accessed 1 April 2023.

[18] De Vries H, Fishta A, Weikert B, Rodriguez Sanchez A (2018). Determinants of sickness absence and return to Work among employees with Common Mental disorders: a scoping review. J Occup Rehabil.

[19] Pevalin DJ, Goldberg DP (2003). Social precursors to onset and recovery from episodes of common mental Illness. Psychol Med.

[20] De Jong K, Nugter MA, Polak MG (2007). The Outcome Questionnaire (OQ-45) in a Dutch population: a cross-cultural validation. Clin Psychol Psychother.

[21] Jacobson NS, Truax P (1991). Clinical significance: a statistical approach to defining meaningful change in psychotherapy research. J Consulting Clin Psychol.

[22] Akay A, Martinsson P. Positional Concerns through the Life Cycle: Evidence from Subjective Well-Being Data and Survey Experiments. Discussion Paper 2012; 6342:1–47.

[23] American Psychiatric Association. Diagnostic and Statistical Manual of Mental Disorders (4th ed., text rev.). 2000.

[24] Lambert MJ, Gregersen AT, Burlingame GM. The Outcome Questionnaire-45. In: Maruish ME, editor. The use of psychological testing for treatment planning and outcomes assessment: Instruments for adults. Lawrence Erlbaum Associates Publishers; 2004. pp. 191–234.

[25] R Development Core Team (2014). R: a Language and Environment for Statistical Computing. R Foundation for Statistical Computing, Vienna, Austria.

[26] Akaike H. Information Theory and an Extension of the Maximum Likelihood Principle. In BN. Petrov, & F. Csaki, Eds. Proceedings of the 2nd International Symposium on Information Theory. Budapest: Akademiai Kiado. 1973;267 – 81.

[27] Bartels SJ, Pratt SI (2009). Psychosocial rehabilitation and quality of life for older adults with serious mental Illness: recent findings and future research directions. Curr Opin Psychiatry.

[28] Paul KI, Scholl H, Moser K, Zechmann A, Batinic B. Employment status, psychological needs, and mental health: Meta-analytic findings concerning the latent deprivation model. Systematic review. Front. Psychol., 02 March 2Sec. Personality and Social Psychology Volume 14–2023 | 10.3389/fpsyg.2023.1017358. 10.3389/fpsyg.2023.1017358 PMC10017486 36935981

[29] Ryan RM, Deci EL (2000). Self-determination theory and the facilitation of intrinsic motivation, Social Development, and well-being January. Am Psychol Am Psychol Association.

[30] Pinquart M, Sörensen S (2003). Differences between caregivers and noncaregivers in psychological health and physical health: a meta-analysis. Psychol Aging.

[31] Vitaliano PP, Scanlon Z, Zhang HM (2003). Is caregiving hazardous to one’s physical health? A meta-analysis. Psychol Bull.

[32] Modini M, Tan L, Brinchmann B, Wang MJ, Killackey E, Glozier N, Harvey SB (2016). Supported employment for people with severe mental Illness: systematic review and meta-analysis of the international evidence. Br J Psychiatry.

[33] Charzyńska K, Kucharska K, Mortimer A (2015). Does employment promote the process of recovery from schizophrenia? A review of the existing evidence. Int J Occup Med Environ Health.

[34] Gammelgaard Wallstroem I, Pedersen P, Nordahl Christensen T (2021). A systematic review of Individual Placement and Support, Employment, and personal and clinical recovery. Psychiatric Serv.

[35] WHO guidelines on mental. health at work https://www.who.int/publications/i/item/9789240053052.

